# The Psychosocial Impact of COVID-19 on an Adult Indian Population

**DOI:** 10.7759/cureus.38504

**Published:** 2023-05-03

**Authors:** Vinita Elizabeth Mani, Rabindra Kumar, Akshat Kumar Srivastava, Zubair Sarkar, Gutti Nagendra Babu, Ruchika Tandon, Vimal Kumar Paliwal, Sanjeev Jha

**Affiliations:** 1 Neurology, Sanjay Gandhi Post Graduate Institute of Medical Sciences, Lucknow, IND; 2 Neurology, Ganesh Shankar Vidyarthi Memorial Medical College, Kanpur, IND

**Keywords:** indian, population, predictors, covid-19, depression, anxiety, psychosocial impact

## Abstract

Background: Coronavirus disease (COVID-19) was a pandemic with many physical, psychological, and socioeconomic effects. COVID-19 caused a global increase in anxiety and depression because of its novelty, high infectivity, varied presentation, and unpredictable mortality. In the face of collapsing healthcare facilities, monetary setbacks, and loneliness because of lockdowns, people were anxious, and this was compounded by media sensationalism. We aimed to study the psychosocial impact of COVID-19 on the adult Indian population.

Methods: An online survey using SurveyMonkey was floated through WhatsApp messages in April 2020, using the ‘chain-referral sampling’ method. Responses from individuals >18 years were included, and questions included age, sex, occupation, demographics, and socioeconomic conditions. The prevalence of anxiety and depression was assessed using the Generalized Anxiety Disorder (GAD-7) and the Patient Health Questionnaire (PHQ-9) scales. Data was analyzed using IBM SPSS software, and predictors of anxiety and depression were assessed.

Results: A total of 2640 responses from individuals between 18 years and 81 years were analyzed, of which 39% were from females and 85% from those <50 years of age. There were students (15.6%), teachers (10.7%), healthcare workers (16.8%), homemakers (9%), and daily wage laborers (4.1%), among others. Nearly 80% lived in cities, 55% had salaried jobs, 37% were working from home, 22% were temporarily unemployed, 10% were feeling work stress, 11% had increased alcohol intake, and 7.5% saw an increase in domestic violence. The income of 50% was adversely affected. Nearly 50% of our respondents had some symptoms of anxiety, and 23% had significant anxiety (GAD ≥5). The presence of anxiety was significantly higher in females, younger adults, city dwellers, healthcare workers, unemployed people, individuals living away from home, those without fixed salaries, those with work stress, and in people whose incomes had been adversely affected by the pandemic. On logistic regression analysis, female sex, younger age, unemployment, lack of salaried jobs, work stress, being a healthcare worker, and media reports were independent predictors of anxiety. About 60% of our respondents had some symptoms of depression, with 26% having significant depression (PHQ-9 ≥5). The presence of depression was significantly higher in females, younger adults, city dwellers, unemployed people, individuals living away from home without fixed salaries, and people with work stress. On logistic regression analysis, younger age, female sex, unemployment, lack of salaried jobs, work stress, and media reports were independent predictors of depression. Among our respondents, 70% used the time during the lockdown to study, 77% caught up with their families, and 56% reconnected with hobbies. Nearly 88% of our respondents had adjusted to their changing circumstances, helped by their religious beliefs and faith, the support of family and friends, good government measures, and the assurance of healthcare.

Conclusions: Significant anxiety and depression were seen in 23% and 26% of respondents, respectively. Being a healthcare worker was an independent predictor of anxiety. Female sex, younger age, unemployment, work stress, and sensational media reports were independent predictors of both anxiety and depression.

## Introduction

Coronavirus disease (COVID-19) has caused unprecedented havoc all around the world, leaving nearly seven million people dead and many more traumatized in its wake [1]. The first case of the novel coronavirus, the severe acute respiratory distress syndrome coronavirus 2 (SARS-CoV-2), was detected in Wuhan, China, towards the end of December 2019. As it rapidly spread around the world, the World Health Organization (WHO) labeled it a pandemic in March 2020. In a short while, countries around the world started imposing ‘lockdowns’ to curtail the spread of the virus, and India followed suit on March 24, 2020. The high infectivity of the novel SARS-CoV-2, lack of herd immunity, inadequate knowledge of its pathophysiology and treatment, unpredictable mortality, extensive media coverage, and inadequate healthcare facilities led to widespread anxiety and depression among people. This was compounded by occupational and monetary setbacks due to the lockdowns, along with loneliness. In other words, it threatened humanity as a whole in all aspects of physical, mental, emotional, social, and economic health.

Anxiety disorders are the most common psychiatric disorders in clinical practice, and generalized anxiety disorder (GAD) is one of the most common anxiety disorders worldwide, with a prevalence of 1.6% to 5% in the general population [2]. The dominant presenting symptoms of GAD include nervousness, trembling, sweating, palpitations, dizziness, and sleep disturbances [3]. Anxiety is often misdiagnosed as depression in outpatient clinics. Major depressive disorder, on the other hand, is characterized by mood abnormalities, loss of interest, guilt, disturbed appetite and/or sleep, low self-worth, tiredness, and impaired concentration, which are episodic and last more than two weeks [4].

Previous pandemics have led to panic globally and have impacted health workers and the general population psychologically, socially, and economically [5]. As the number of COVID-19-positive cases surged in our country, the number of those directly and indirectly affected by the disease also increased. This study was conducted to assess the psychological and socioeconomic impact of the COVID-19 pandemic on the adult Indian population.

## Materials and methods

Aims and objectives

We aimed to study the psychological and socioeconomic impact of COVID-19 on adult Indians, focusing on the prevalence of anxiety and depression. In addition, we also aimed to evaluate the predictors of anxiety and depression in our study population.

Study design

An online survey questionnaire using SurveyMonkey software was floated among the general population using the WhatsApp platform from 25th April to 10th May 2020. Both English and Hindi versions of the survey were floated simultaneously. The ‘chain-referral sampling method’ was used, in which each respondent was asked to forward the questionnaire to multiple people, who were expected to continue the process. 

Ethical approval 

The Institute Ethics Committee Sanjay Gandhi Post Graduate Institute of Medical Sciences, Lucknow, approved this study. (IEC code: 2020-128-IP-EXP-18).

Target population

The survey was open to all adult Indians over the age of 18 years. 

Exclusion criteria

Respondents with pre-existing mental illnesses were excluded from the study. Responses that were incomplete with regards to consent, anxiety and depression scales, age, and sex were also excluded from the study.

Survey questionnaire

All respondents had to read an informed consent and click on ‘agree’ or ‘disagree’ before proceeding. The questionnaire was designed to collect data regarding age, sex, educational status, occupation, geographical state of residence, and the presence of psychiatric illness and other comorbidities. Other data included the type of income (salary, contractual, or daily wage) and whether income had been adversely affected due to the pandemic. Respondents were asked whether media reports had added to their anxiety or not. Questions regarding the ability of the respondents to adjust to their changing circumstances were also included, and a comment box was provided at the end for individual opinions. 

Assessment tools for anxiety and depression

We used the Generalized Anxiety Disorder-7 (GAD-7) as a self-administered screening tool for anxiety and the Patient Health Questionnaire-9 (PHQ-9) similarly for depression. Both GAD-7 and PHQ-9 are validated as reliable tools and have been extensively used for the screening and severity stratification of GAD and depression, respectively [6,7]. 

The GAD-7 consists of seven items, each of which is scored from 0-3 depending on how the subjects have been affected by them in the preceding two weeks: 0: not at all, 1: several days, 2: more than half the days, 3: nearly every day. The total score ranges from 0 to 21, and subjects are classified into mild, moderate, and severe anxiety if scores range between 5-9, 10-14, and 15-21, respectively [6]. We additionally classified respondents as having minimal anxiety if their GAD-7 scores ranged between 1 and 4.

The PHQ-9 has nine items, each of which is scored from 0-3 depending on how the subjects have been affected by them in the preceding two weeks: 0: not at all, 1: several days, 2: more than half the days, 3: nearly every day. The total score ranges from 0 to 27, and subjects are classified into mild, moderate, moderately severe, and severe depression if scores range between 5-9, 10-14, 15-19, and ≥ 20, respectively [7]. We additionally classified subjects as having minimal depression if their PHQ-9 scores ranged between 1 and 4. 

Statistical analysis

All data were analyzed using IBM SPSS Statistics for Macintosh, Version 26.0 (Armonk, NY: IBM Corp.). Descriptive statistics with frequency analysis were used for categorical variables, and relationships between categorical variables were established using the Chi-square test or the Fisher exact test. A two-tailed p-value <0.05 was considered statistically significant. The predictors of anxiety and depression were assessed using logistic regression analysis.

## Results

**Figure 1:**
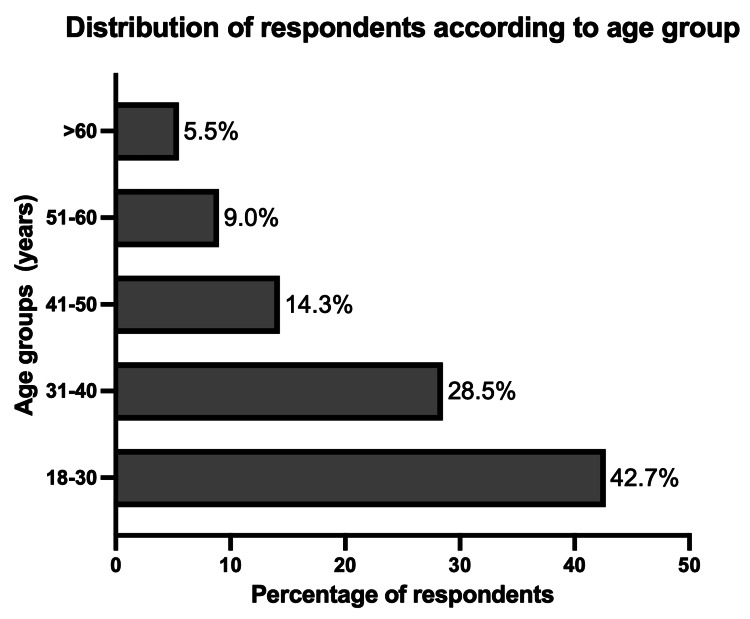
Distribution of respondents according to age group

There were 3093 responses, of which 453 were discarded from analysis due to incomplete entries for demographic data or anxiety and depression scales. A total of 2640 respondents, ranging in age from 18 to 81 (mean 35.91 ± 13.16 years), completed the survey. There were 1609 males (61%) and 1031 females, and 85% (2251/2640) of them were younger than 50 years, with only 155 persons aged more than 60 years. Figure 1 shows the distribution of our respondents according to age groups. Most of the respondents were from North India (1794/2640; 68%), though there was representation from every zone of the country, with 325 from the south, 298 from the west, 138 from the east, 68 from central India, and 17 from the northeast zone. Eighty percent of the survey participants were residing in towns and cities at the time of the survey.

**Figure 2:**
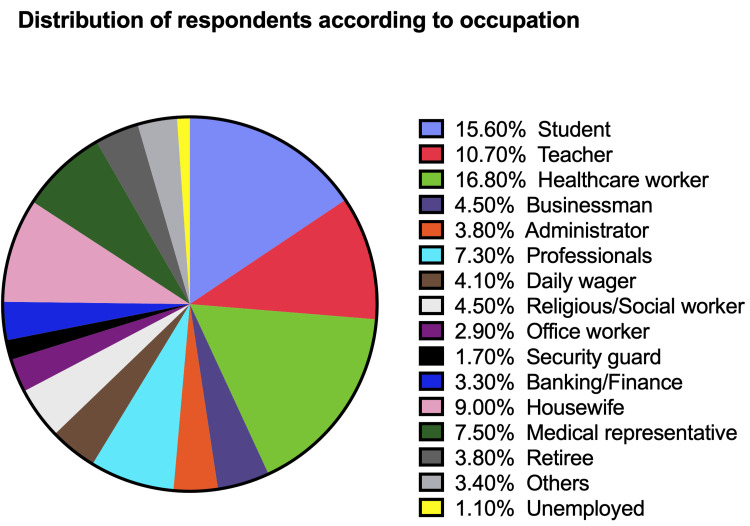
Distribution of respondents according to occupation

Among the 2640 respondents, 15.6% (412) were students, 282 (10.7%) were teachers, and 443 (16.8%) were healthcare workers. Besides, we had businessmen (4.5%), administrators (3.8%), professionals consisting of lawyers, information technologists, and engineers (7.3%), daily wage laborers (4.1%), religious leaders, and social workers (4.5%), office workers (2.9%), bankers (3.3%), homemakers (9%), medical representatives (7.5%), security guards (1.7%), retired personnel (3.8%), and even some who were unemployed (1.1%). Figure 2 shows the distribution of our respondents according to their occupation. Nearly 95% of the respondents had completed high school, 893 (34%) were graduates, 782 (30%) had a postgraduate degree, and 314 (12%) had or were pursuing a doctorate. 

Though most respondents were living at home (85%), 402 (15%) were stranded away from home due to the lockdown, were in hostels, or were living in slums. A total of 299 (11.3%) of the respondents had co-morbidities requiring medications. One-fifth of the respondents (21%) said access to healthcare was difficult. This difficulty was more pronounced in the rural setting than in cities (41% vs. 16%; p <0.001).

Some respondents, like students and homemakers (26%), had no source of income, but 55% of the respondents had regular salaried jobs, 222 (8%) were in contractual jobs, 106 (4%) had businesses with irregular incomes, and 174 (7%) were daily wage laborers. 

The psychological effects of COVID-19

Anxiety and Its Predictors

**Figure 3:**
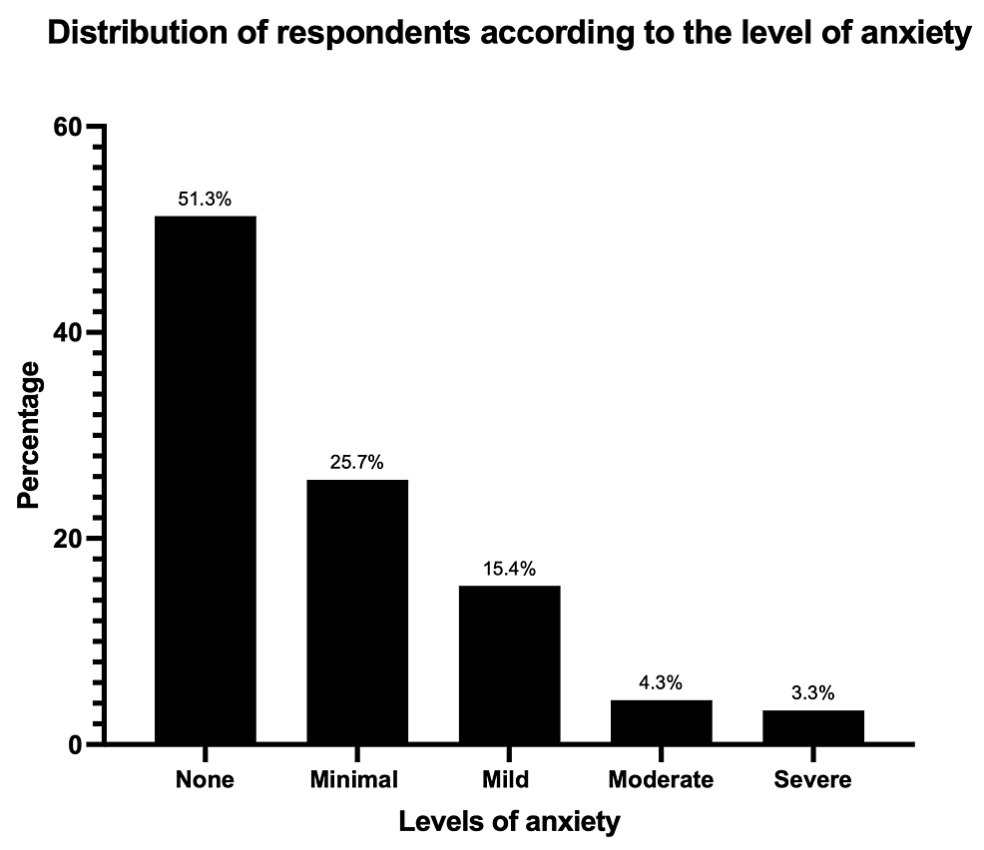
Distribution of respondents according to the level of anxiety

About 50% of our respondents (1286/ 2640) had some symptoms of anxiety. Their GAD-7 scores ranged between 0 and 21 (mean score 2.71 ± 4.33), with 26% having minimal anxiety (GAD-7 scores 1-4). A total of 608 (23%) respondents had significant anxiety (GAD-7 score ≥5), of which 407 (67%) had mild anxiety, 114 (19%) had moderate anxiety, and 87 (14%) had severe anxiety. Figure 3 shows the distribution of our respondents according to their level of anxiety.

Subjects older than 50 years were less anxious than those who were younger (17% vs. 24.1%; p 0.002). Females were more anxious than males (27.6% vs. 20.1%; p <0.001), and the urban population was more anxious than those living in villages (25% vs. 15.9%; p <0.001). Healthcare workers were more anxious than others (28.2% vs. 22%; p 0.005), and those living away from their homes were also more anxious (28.4% vs. 22.6%; p 0.02). Anxiety was significantly higher in people without salaried jobs (26.8% vs. 20.5%; p <0.001), those who were unemployed (33% vs. 20.2%; p <0.001), and those experiencing stress at work (42.2% vs. 20.8%; p <0.001). A significantly higher number of people with anxiety also had increased alcohol intake (39.4% vs. 21.1%; p <0.001) and more domestic violence in their homes (45.2% vs. 21.2%; p <0.001). Respondents whose income was adversely affected were more anxious (26.7% vs. 19.5%; p <0.001), as were those who were unable to adjust to their changing circumstances (30.1% vs. 21.2%; p 0.002). Regular media updates and sensationalism added to the anxiety of 44% of the respondents. Table 1 shows the demographic and socioeconomic factors affecting anxiety in our respondents.

**Table 1:** Impact of various demographic and socioeconomic factors on anxiety (GAD-7 ≥5)

S. No	Demographic and socioeconomic factors				Demographic and socioeconomic factors				P value
	Factors	Total no. of respondents	No. of respondents with anxiety	Percentage of respondents with anxiety	Factors	Total no. of respondents	No. of respondents with anxiety	Percentage of respondents with anxiety	P value
1.	Age > 50 years	389	66	17%	Age < 50 years	2251	543	24.1%	0.002
2.	Males	1609	324	20.1%	Females	1031	284	27.6%	<0.001
3.	Urban	2110	125	25.0%	Rural	509	81	15.9%	<0.001
4.	Healthcare worker	443	125	28.2%	Others	2197	483	22.0%	0.005
5.	Education > post graduate	1090	270	24.8%	Education < post graduate	1518	334	22.0%	0.10
6.	Away from home	363	103	28.4%	At home	2076	469	22.6%	0.02
7.	Salaried job	1413	289	20.5%	Non-salaried job	1155	309	26.8%	<0.001
8.	Income affected	1287	344	26.7%	Income not affected	1353	264	19.5%	<0.001
9.	Stressed by media reports	1163	430	37.0%	Not stressed by media reports	1467	175	11.9%	<0.001
10.	Alcohol abuse	282	111	39.4%	No alcohol abuse	2358	497	21.1%	<0.001
11.	Domestic violence	199	90	45.2%	No domestic violence	2441	518	21.2%	<0.001
12.	Work stress	270	114	42.2%	No work stress	2370	494	20.8%	<0.001
13.	Temporarily unemployed	575	190	33.0%	Employed	2065	418	20.2%	<0.001
14.	Able to adjust	1757	373	21.2%	Unable to adjust	246	74	30.1%	0.002

On binary logistic regression, younger age (OR 1.35; p 0.05), female sex (OR 1.40; p 0.001), healthcare workers (OR 1.49; p 0.003), lack of salaried jobs (OR 1.27; p 0.03), unemployment (OR 2.02; p <0.001), work stress (OR 2.53; p <0.001) and media reports (3.94; p <0.001) were found to be independent predictors of anxiety.

Depression and Its Predictors

**Figure 4:**
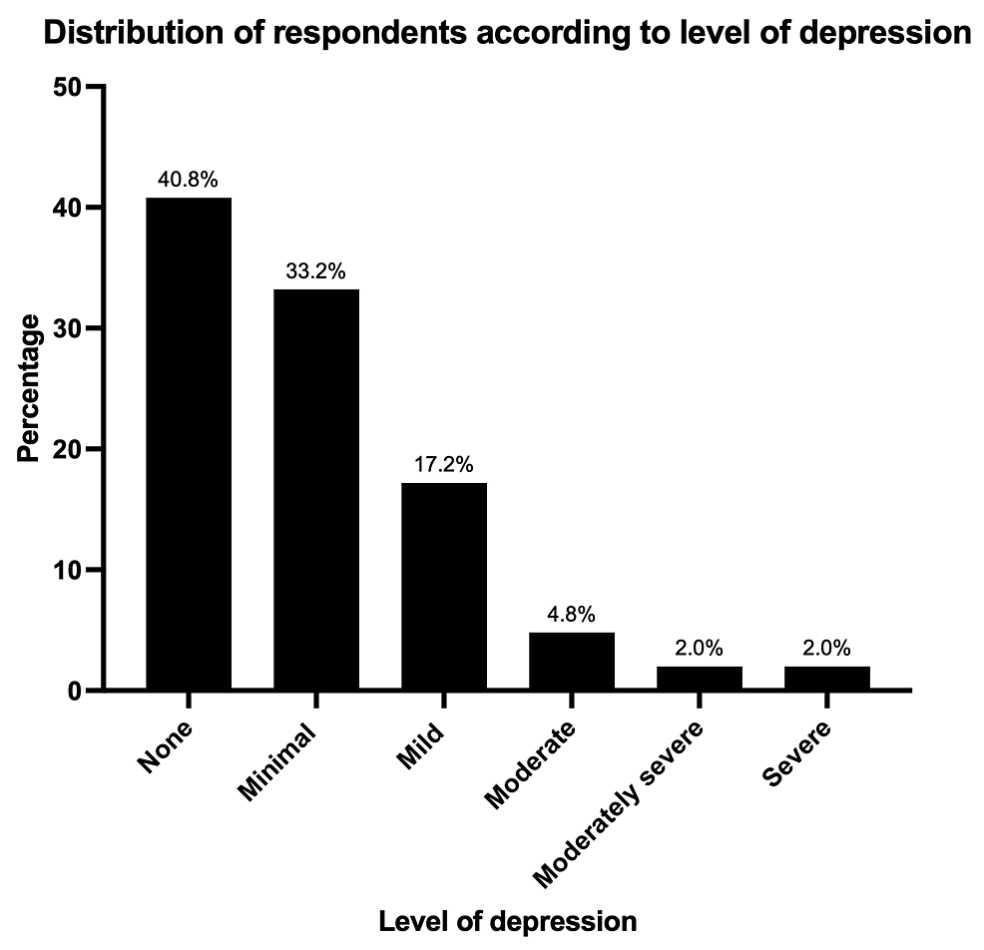
Distribution of respondents according to the level of depression

The PHQ-9 scores of our respondents ranged between 0 and 27 (mean 3.31 ± 4.83). Nearly 60% of the respondents (1566/2640) expressed some depressive symptoms on the PHQ-9, with 33% (876) having minimal depression (PHQ-9 scores 1-4). A total of 690 (26%) respondents had clinically significant depression (PHQ-9 score ≥5), of which 455 (65.9%) had mild depression, 128 (18.6%) had moderate depression, 53 (7.7%) had moderately severe depression, and 54 (7.8%) had severe depression. Figure 4 shows the distribution of our respondents according to their level of depression.

Females were more depressed than males (29.6% vs. 23.9%; p <0.001). People younger than 50 years were more depressed (27.6% vs. 17.7%; p <0.001), and people living in cities were more depressed than those from rural areas (27.7% vs. 19.8%; p <0.001). Those who were living away from their home were more depressed (32% vs. 25.5%; p 0.01), as were those whose incomes were affected due to COVID-19 (30.4% vs. 22.1%; p <0.001), those without a regular salary (30.4% vs. 23.2%; p <0.001) and those who were unemployed (37.4% vs. 23%; p <0.001). Though a higher percentage of healthcare workers were depressed than the general population, this difference was not statistically significant (29.8% vs. 25.4%; p 0.06). More people felt stressed at work (39.6% vs. 24.6%; p <0.001), and those unable to adjust to their circumstances (37.8% vs. 23.7%; p <0.001) were depressed. Alcohol abuse and an increase in domestic violence were seen in a significantly higher number of people with depression (45.4% vs. 23.8%; p <0.001 and 51.3% vs. 24.1%; p <0.001, respectively). Table 2 shows the various demographic and socioeconomic factors affecting depression in our respondents.

**Table 2:** Impact of demographic and socioeconomic factors on depression (PHQ-9 ≥5)

S. No	Demographic and socioeconomic factors				Demographic and socioeconomic factors				P value
	Factors	Total no. of respondents	No. of respondents with depression	Percentage of respondents with depression	Factors	Total no. of respondents	No. of respondents with depression	Percentage of respondents with depression	P value
1.	Age > 50 years	389	69	17.7%	Age < 50 years	2251	621	27.6%	<0.001
2.	Males	1609	385	23.9%	Females	1031	305	29.6%	<0.001
3.	Urban	2110	584	27.7%	Rural	509	101	19.8%	<0.001
4.	Healthcare worker	443	132	29.8%	Others	2197	558	25.4%	0.06
5.	Education > post graduate	1090	288	26.4%	Education < post graduate	1518	395	26.0%	0.82
6.	Away from home	363	116	32.0%	At home	2076	529	25.5%	0.01
7.	Salaried job	1413	328	23.2%	Non salaried job	1155	351	30.4%	<0.001
8.	Income affected	1287	391	30.4%	Income not affected	1353	299	22.1%	<0.001
9.	Stressed by media reports	1163	439	37.8%	Not stressed by media reports	1467	250	17.0%	<0.001
10.	Alcohol abuse	282	128	45.4%	No alcohol abuse	2358	562	23.8%	<0.001
11.	Domestic violence	199	102	51.3%	No domestic violence	2441	588	24.1%	<0.001
12.	Work stress	270	107	39.6%	No work stress	2370	583	24.6%	<0.001
13.	Temporarily unemployed	575	215	37.4%	Employed	2065	475	23.0%	<0.001
14.	Able to adjust	1757	416	23.7%	Unable to adjust	246	93	37.8%	<0.001

After binary logistic regression analysis, we found that younger age (OR 1.59; p 0.002), female sex (OR 1.25; p 0.02), lack of salaried jobs (OR 1.24; p 0.04), unemployment (OR 1.94; p <0.001), work stress (OR 1.91; p <0.001), and media reports (OR 2.70; p <0.001) were independent predictors for depression in our population.

The socioeconomic effects of COVID-19

Eighty-six percent (2270/2640) of the respondents admitted that their work was affected in some way or another. A total of 975 (37%) respondents were forced to work from home, and 22% were temporarily unemployed. Only 10% said they were more relaxed while working from home, while 10% were feeling stressed from being overworked at their jobs. The income of nearly half (1287/2640) of the respondents was affected due to the restrictions imposed on account of COVID-19. About 282 (11%) respondents said that their alcohol intake had increased, and 199 (7.5%) saw an increase in domestic violence. Nearly 32% (846) of the respondents said they were hoarding groceries and medical supplies at home. 

A few positive outcomes: the rainbow after the storm

More than 70% of respondents (1831/2640) used the time during the lockdown to study, while 1453 (56%) caught up on hobbies. About 77% of people said that the pandemic had brought their family members closer to each other, and 75% (1948) said that it had made them more religious. Nearly 88% (1757) of the respondents were able to adjust to the drastic changes in lifestyle at the time of the survey. Some of the factors that helped people adjust to the changing circumstances were their religious beliefs and faith in God (76%), family support (70%), a strong friend group (41%), good government measures (35%), and assurance of health care (31%).

## Discussion

COVID-19, with its high infectivity and unpredictable mortality, led to mass panic, ‘coronaphobia’ and economic losses for individuals and countries, leading to myriad psychosocial manifestations worldwide [8]. According to the data released by the COVID-19 Mental Disorders Collaborators, the COVID-19 pandemic caused a global increase in cases of anxiety disorder by 26% and major depressive disease by 28% [9]. Our study on the Indian population showed similar findings, with 23% having symptoms of significant anxiety and 26% having symptoms of depression.

Our study results showed that females and respondents younger than 50 years were significantly more anxious and depressed than males. A study of Palestinian university students showed that 78% of them had symptoms of anxiety [10]. A systematic review of the global burden of anxiety and depression due to COVID-19 also found that both anxiety and depression were more common in younger people and females [9].

Our respondents had varied occupations, with students, teachers, businessmen, administrators, lawyers, engineers, bankers, office workers, religious leaders, homemakers, security guards, retirees, and unemployed people, besides healthcare workers, who constituted only 16.8% of the total respondents. This is in contrast to a study on anxiety due to COVID-19 among Indians, in which nearly 50% of respondents were healthcare workers. It was a smaller study (662 respondents) on highly educated (90% graduate and above) English-speaking people, who are a minority in the Indian subcontinent [11]. Their study population was also younger (mean age 29.09 ± 8.83 years) [11] when compared to ours, in which the mean age was 35.91 ± 13.16 years, and our oldest participant was 81 years of age.

A large nationwide survey from China revealed that migrant workers had the highest levels of distress [12]. These results were echoed in our study, where people stranded away from home during the lockdown were significantly more anxious and depressed than others living at home. This could be attributed to the lack of proper accommodation, finance, provisions, and family support during the stressful lockdown period.

In our study, 44% of the respondents said that media reports contributed to their anxiety. This finding is corroborated by Roy et al.’s study, which found half of their respondents anxious because of social media [11]. The ‘coronavirus infodemic’ propagated by social media included a lot of useful information but also created panic with fake information, rumors, and sensationalism [13]. In the study by Roy et al., 33% of their 662 respondents stocked up on groceries and essentials, which is very similar to our study, where 32% felt it necessary to stock up on provisions and medicines [11]. Panic buying is often seen in pandemics and other natural disasters, which leads to a scarcity of resources, which further adds to the anxiety and depression.

A study on female nursing students from Saudi Arabia found anxiety in 24% and depression in 19%, and documented that family income, family support, the presence of chronic illness, and exposure to COVID-19 were predictors of anxiety, while family income, family support, and a history of mental illness were predictors of depression [14]. In a Turkish survey, female sex, living in cities, and a history of psychiatric illness were significant predictors of anxiety, while urban living was a significant predictor of depression [15]. In a study among university students in Malaysia, family income was a predictor of anxiety, while female gender was a predictor of depression [16]. A study from China in the early phases of the pandemic showed high levels of anxiety, depression, insomnia, and stress among frontline health workers, especially nurses [17]. Another study on frontline healthcare workers from Brazil also showed significant levels of anxiety and depression, which were higher in female health workers than males [18]. Our study showed that female sex, unemployment, a lack of salaried jobs, work stress, and media sensationalism were independent predictors of both anxiety and depression among the Indian people. Being a healthcare worker was an independent predictor of anxiety in our study population.

With time, most people adapt psychologically to traumatic and stressful situations. In our study, we saw that many of our respondents constructively used their time at home during the lockdown to study, reconnect with family and friends, pursue their hobbies, and engage in religious activities. Another survey from Trinidad and Tobago during the pandemic also had very similar results [19]. A study from the United Kingdom during the pandemic revealed that engaging in leisure activities and creative crafts predicted well-being among people [20]. Various factors, like the availability of medical facilities and public health resources, are known to influence psychological distress during epidemics and pandemics [21]. In our study, faith in God and support from family and friends were also some of the factors that, along with the assurance of medical care and government measures, helped respondents adjust to the unpredictable and changing times.

Strengths of our study 

This is one of the largest surveys on the psychosocial impact of COVID-19 among Indians. We had a large number of respondents from the Indian subcontinent, with representation from nearly all strata of education, occupation, and economic backgrounds. We have used the GAD-7 and PHQ-9 scales, which are validated tools for self-assessment of anxiety and depression, respectively. We have also calculated the predictors for anxiety and depression in our population. Besides, we have addressed the socioeconomic impact of COVID-19 and highlighted a few positive outcomes of the pandemic.

Limitations of the study

Though we had respondents from all zones of the country, the maximum number of responses were from North India, with relatively few responses from other zones. So this data may not be truly representative of the entire country, which boasts a rich diversity of cultures. As this was an online survey, only those with access to smartphones could participate, limiting it largely to those who are younger and live in towns and cities. Elderly people often find technology challenging, which limits their participation in online surveys. Internet availability could also have been a problem in villages, accounting for fewer responses from rural areas.

## Conclusions

According to our study, half of our respondents had some symptoms of anxiety, and 23% of our population had significant anxiety. Besides, nearly two-thirds had some symptoms of depression, with 26% having clinically significant depression. Females and younger people were significantly more anxious and depressed than males and older people. Healthcare workers were also significantly more anxious than the general population. Female sex, unemployment, lack of salaried jobs, healthcare workers, work stress, and media reports were independent predictors of both anxiety and depression.

If we were to extrapolate our data onto India’s massive population, we can safely say that in the Indian subcontinent alone, millions of people were anxious and/or depressed during the first wave of COVID-19. This clearly shows that individual and population mental health was severely compromised due to the pandemic, and measures need to be taken to promote psychological and socioeconomic well-being along with physical well-being.
